# Creation of Polymer Hydrogelator/Poly(Vinyl Alcohol) Composite Molecular Hydrogel Materials

**DOI:** 10.3390/gels9090679

**Published:** 2023-08-23

**Authors:** Yutaka Ohsedo, Wakana Ueno

**Affiliations:** 1 Division of Engineering, Faculty of Engineering, Nara Women’s University, Kitauoyahigashi-Machi, Nara 630-8506, Japan; 2 Faculty of Human Life and Environment, Nara Women’s University, Kitauoyahigashi-Machi, Nara 630-8506, Japan

**Keywords:** polymer hydrogelator, molecular hydrogel, thixotropic behavior, poly(vinyl alcohol), composite

## Abstract

Polymer hydrogels, including molecular hydrogels, are expected to become materials for healthcare and medical applications, but there is a need to create new functional molecular gels that can meet the required performance. In this paper, for creating new molecular hydrogel materials, the gel formation behavior and its rheological properties for the molecular gels composed of a polymer hydrogelator, poly(3-sodium sulfo-*p*-phenylene-terephthalamide) polymer (**NaPPDT**), and water-soluble polymer with the polar group, poly(vinyl alcohol) (**PVA**) in various concentrations were examined. Molecular hydrogel composites formed from simple mixtures of **NaPPDT** aqueous solutions (0.1 wt.%~1.0 wt.%) and **PVA** aqueous solutions exhibited thixotropic behavior in the relatively low concentration region (0.1 wt.%~1.0 wt.%) and spinnable gel formation in the dense concentration region (4.0 wt.%~8.0 wt.%) with 1.0 wt.% **NaPPDT** aq., showing a characteristic concentration dependence of mechanical behavior. In contrast, each single-component aqueous solution showed no such gel formation in the concentration range in the present experiments. No gel formation behavior was also observed when mixed with common anionic polymers other than **NaPPDT**. This improvement in gel-forming ability due to mixing may be due to the increased density of the gel’s network structure composed of hydrogelator and **PVA** and rigidity owing to **NaPPDT**.

## 1. Introduction

Gels encompass old and new forms of substances and materials that are typically found in food and other aspects of life [1]. These gel-like substances are known to be formed from various materials such as organic polymers and clay minerals; such substances that are formed from polymers are typically regarded as polymer gels [2,3]. Polymer gels contain solvents in the three-dimensional network structure of polymer chains, and there is interest in academic investigations aimed at elucidating the correlation between material properties and the polymer structure [2,3]. Conversely, by designing and controlling the interaction between polymer chains or solvent molecules inside a polymer gel, or by designing and controlling the network structure, it is possible to introduce various functionalities, including external stimulus responsiveness. Thus, there is interest in the development and application of intelligent functions such as sensors and actuators, and active research and development is underway [4]. In particular, polymer hydrogels, which are hydrogels and polymer gels, are expected to have an affinity with living organisms, as their structure is similar to that of cells, the extracellular matrix, and other biomaterials because the living organism itself can be considered a hydrogel. For these reasons, polymer hydrogels are being actively investigated as polymeric biomaterials that can be used as cell scaffold materials, drug delivery systems, or artificial organs, and their development is attracting attention from both the basic and applied perspectives [4,5,6,7]. In applications other than biomaterials, polymer gels have attracted attention as a form of functional polymeric material, and various research and development efforts are underway for their practical applications. For example, gel actuators that realize macroscopic movements using polymer hydrogels are attracting attention as a material that can lead to the creation of artificial muscles made from polymer hydrogels in the future because of the smooth and soft movements derived from gel-like substances and the expected biocompatibility of polymer hydrogels which is expected to be applied in the medical field [8]. As described above, polymer gels, especially polymer hydrogels, are one of the material forms and gel materials that have been actively focused on in recent years for the creation of new materials in terms of both basic science and applications. Among various polymer gels, molecular gels composed of polymer gelators have received remarkable attention for research and application due to their ease of acquisition, as gels can be reproducibly obtained simply by standing a solution obtained from thermal dissolution in a solvent or room-temperature dissolution [9,10,11,12,13,14].

We have focused on molecular gels that form self-assembled or cohesive fiber structures and are physically crosslinked by reversible interactions between molecules, and as part of our research on the fabrication of new molecular gel materials, we have conducted a series of studies on the function of water-soluble aromatic polyamide poly(3-sodium sulfo-*p*-phenylene-terephthalamide) (**NaPPDT**) [15] as a polymer hydrogelator. The hydrogel obtained from this gelator possesses a thixotropic property [16,17,18], which is of considerable interest as a required essential property for creams and ointments, as it exhibits a reversible change from a sol state to a gel state when subjected to an external mechanical force. Furthermore, this polymer hydrogelator is a thixotropic composite gel material that serves as a matrix for various organic and inorganic materials, such as inorganic nanosheet Laponite [19] and water-dispersible polyanilines [20,21], both of which have potential applications as a base material for ointments in the healthcare fields [22,23,24,25].

**Figure 1:**
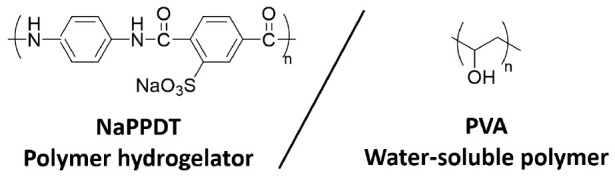
Chemical structures of polymer hydrogelator **NaPPDT** and water-soluble polymer **PVA**.

Herein, to fabricate novel molecular gel materials using polymer gelators as a matrix in healthcare fields, a new composite molecular hydrogel is fabricated by mixing a water-soluble polymer with **NaPPDT** (Figure 1). Poly(vinyl alcohol) (**PVA**) was selected as the water-soluble polymer to be mixed, as it is an electrostatically neutral non-ionic water-soluble polymer that is not expected to form water-insoluble polyionic complexes with **NaPPDT** containing a sodium sulfonate salt moiety, and it is a proven compound as a hydrophilic component in numerous polymer composites [26,27]. In the fabrication of composite gels by mixing polymers with gelling agents, the behavior of gel formation may be tuned by the molecular weight of the polymers. In this study, simple mixing of **NaPPDT** and **PVA** aqueous solutions at room temperature resulted in composite molecular gels that exhibit different mechanical properties compared to **NaPPDT** molecular hydrogels. This study revealed a thixotropic hydrogel material with a novel polymeric network structure, which is not observed in a single system of the same concentration region and is inferred to have a new polymeric network structure, as shown below.

## 2. Results and Discussion

**Figure 2:**
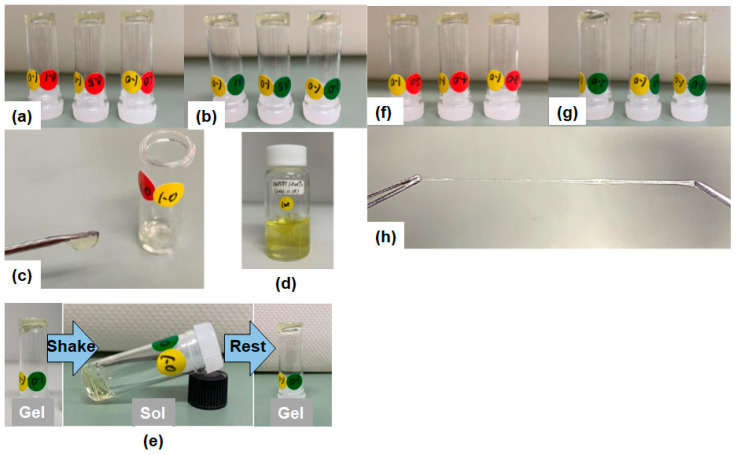
Gelation and thixotropic behavior of mixed composite **NaPPDT**/**PVA** 1/1 (*w*/*w*) molecular gel systems (concentration ratios shown as “wt.%/wt.%”): (**a**) **NaPPDT**/**PVA-L** system, left: 1.0/0.1, middle: 1.0/0.5, right: 1.0/1.0; (**b**) **NaPPDT**/**PVA-H** system, left: 1.0/0.1, middle: 1.0/0.5, right: 1.0/1.0; (**c**) thixotropic behavior of the scooped composite gel **NaPPDT**/**PVA-L** 1.0/1.0; (**d**) **NaPPDT** 1.0 wt.%; (**e**) **NaPPDT**/**PVA-H** 1.0/1.0 (shaken and sol, then left to stand for 1 min and recovered to gel state); (**f**) **NaPPDT**/**PVA-L;** left: 1.0/2.0; middle: 1.0/4.0; right: 1.0/8.0; (**g**) **NaPPDT**/**PVA-H**; left: 1.0/2.0; middle: 1.0/4.0; right: 1.0/4.0; right: 1.0/8.0; (**h**) pulled spinnable gel obtained from **NaPPDT**/**PVA-H** 1.0/8.0.

First, the mixing of the prepared **NaPPDT** aqueous solutions with **PVA** aqueous solutions at room temperature by vortex mixer and the resulting mixed solution were observed (Table 1 and Figure 2). The polymer hydrogelator **NaPPDT** was a sample obtained by polycondensation, according to a previous study [15]. The **NaPPDT** solution is the liquid state at 0.5 wt.%, but at 1.0 wt.%, the solution has good thixotropic properties with a recovery time to the gel state within 1 min after making sol by applying external force. The resulting mixed solution is a light-yellow molecular hydrogel with good thixotropic properties and a recovery time to gel within 1 min after solvation under external force. As **NaPPDT** at concentrations above 2.0 wt.% does not flow by pressing and requires time to mix with other components, aqueous solutions with **NaPPDT** concentrations of 0.5 wt.% (liquid) and 1.0 wt.% (gel) were used for mixing with the aqueous **PVA** solution. The adjusted aqueous **PVA** solution became viscous at 8.0 wt.%, and as mixing with aqueous **NaPPDT** solution by simple vortex mixing would be difficult at higher concentrations, a concentration series was prepared with the highest concentration of 8.0 wt.% for the **PVA** solutions. Aqueous **NaPPDT** solutions (which form a gel at ≥1.0 wt.%) and aqueous **PVA** solutions (which were in a solution state with no gel formation even at 8.0 wt.% for both types of **PVA** described below) were mixed at a weight ratio of 1:1, and the gel-forming ability of the mixtures was evaluated (low and high molecular weight PVAs were designated **PVA-L** and **PVA-H**, respectively, as shown in Table 1); the mixture of 1.0 wt.% **NaPPDT** formed a gel in both combinations, although the concentration of **NaPPDT** after mixing was 0.5 wt.%, which is not gel-forming, indicating that the complexes were formed by the interaction of **PVA** with **NaPPDT**, which is involved in gel formation. The resulting gel could be extracted and showed thixotropic properties.

To determine whether hydrogel formation by mixing aqueous **NaPPDT**/**PVA** systems in this low concentration range is a common phenomenon observed in the mixing of other anionic polymer/**PVA** systems other than **NaPPDT**, with other sodium sulfonates or phosphoric acids as side-chain polar groups of each of the polymers (1.0 wt.%) used, i.e., poly(sodium 4-styrenesulfonate) (two polymers with different molecular weights), chondroitin sulphate sodium salt, and deoxyribonucleic acid sodium salt, 1.0 wt.% aqueous polymer solutions were mixed with 1.0 wt.% aqueous **PVA** solutions and observed (Table 2). The results showed that the mixture was a clear solution and did not form a gel, indicating that the thixotropic gel formation due to earlier mixing in the low concentration range is a characteristic result of the **NaPPDT**/**PVA** complex. This is presumably because a certain degree of main chain rigidity is beneficial for gel formation at low concentrations, and other nonconjugated polymers with sodium sulfonate or phosphoric acid as side-chain polar groups lack rigidity or concentration. The mixing of higher concentrations of **PVA** solutions from 2.0 wt.% to 8.0 wt.% with a 1.0 wt.% **NaPPDT** solution was then investigated (Table 1). The results showed that, as with the mixing in the low concentration range, gels were obtained by mixing, but when the **PVA** concentration was above 4.0 wt.%, the gels obtained showed spinnability not seen in **PVA** alone systems of the same concentration, indicating that spinnable hydrogels were obtained (Figure 2h). These results also suggest that complexation of **PVA** and **NaPPDT** by mixing is involved in gel formation and that complexation by mixing is involved in the development of the spinnability of the gels obtained.

**Figure 3:**
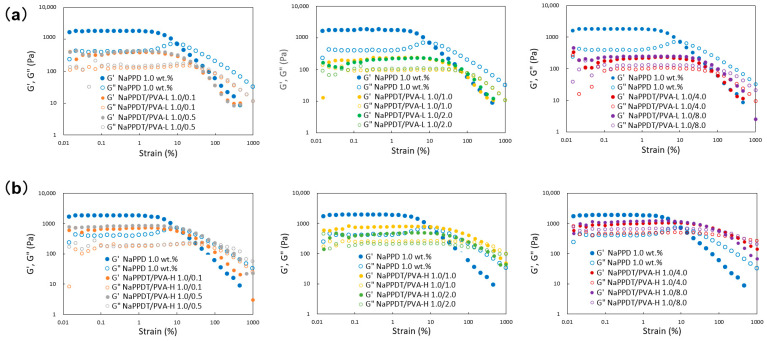
Dynamic rheological properties of the mixed composite **NaPPDT**/**PVA** 1/1 (*w*/*w*) molecular gel systems on strain sweep: (**a**) **NaPPDT**/**PVA-L** systems; (**b**) **NaPPDT**/**PVA-H** systems.

To quantitatively evaluate the composite gel formation described above, dynamic viscoelasticity measurements (strain dispersion measurements) were performed using a rheometer, and the results are shown in Figure 3. In both systems, the composite gels were gels that transitioned from G′ > G″ (gel) to G′ < G″ (sol) [28] and had a lower modulus than **NaPPDT** alone at all **PVA** concentrations, and they are softer than **NaPPDT** alone at all **PVA** concentrations. The elastic modulus tended to increase with increasing **PVA** concentration, and the transition strain increased compared with the **NaPPDT** alone system, making the gels more resistant to deformation. In contrast, the **NaPPDT**/**PVA-H** system produced a stable gel with a higher modulus than the **PVA-L** system (less blurring of the measurement plots). The elastic modulus in the region above 100% strain was greater than that of the **NaPPDT** alone system. This shows that the higher molecular weight of **PVA** may have improved the gel network to the extent that it exhibited a high elastic modulus, resulting in the development of towing properties from gel formation. There was also an interesting trend toward higher modulus but lower transition strain at **PVA** concentrations that resulted in spinnable gels. The details of this phenomenon are a subject for further research, but it may be related to the gel’s spinnability and ability to expand.

**Figure 4:**
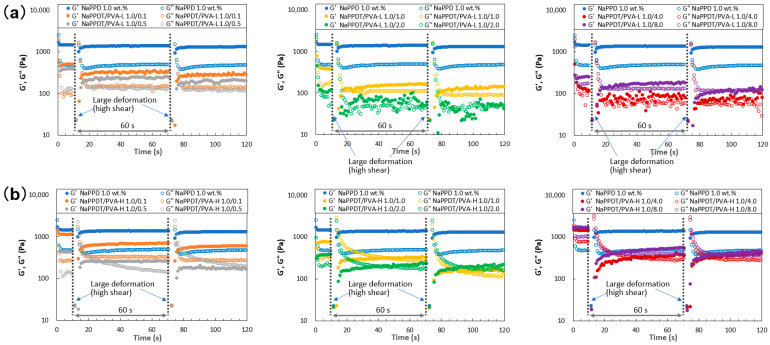
Thixotropic behavior of the mixed composite **NaPPDT**/**PVA** 1/1 (*w*/*w*) molecular gel systems (concentration ratios shown as wt.%/wt.%): (**a**) **NaPPDT**/**PVA-L** systems; (**b**) **NaPPDT**/**PVA-H** systems.

Next, the thixotropic properties of the obtained composite gels were quantitatively evaluated using dynamic viscoelasticity measurements. The results shown in Figure 4 indicate that both composite hydrogels in both **PVA-L** and **PVA-H** systems exhibit reversible recovery from gel to sol after significant deformation. The degree of recovery, as determined by comparing the modulus of elasticity, showed that they had almost returned to their pre-deformation state. A trend from G′ > G″ to G′ ≥ G″ was observed after gel recovery as the **PVA** solution concentration increased in both systems. The scatter of the elastic modulus plots and the shape stability indicate that the high molecular weight **PVA-H** is more stable than **PVA-L**. This suggests that the high molecular weight **PVA-H** has an advantage in recovering to the gel state after large deformation. This may indicate that the presence of high molecular weight **PVA** is more effective in inter-network linkages in the recovery behavior and macroscopic recovery of the gel state. The reason for this could be that higher molecular weight, longer molecular chain lengths, and larger average radii of polymer chain filaments are more favorable for inter-network linkages compared to lower molecular weight polymers. However, although the **PVA-H** composite gels recovered repeatedly, at higher **PVA** concentrations the distance between G′ and G″ was almost the same as at low **PVA** concentration. This is probably due to the predominance of the **NaPPDT** property at low **PVA** concentrations, but the predominance of the liquid property of **PVA** as the **PVA** concentration increases; spinnable gels were obtained at high **PVA** concentrations, but the spinnable property is close to liquid and gel may be important. This may be because the plots were stable in a stable gel state, but as the **PVA** concentration increased and the liquid properties appeared, the plots became unstable and blurred. This trend was more pronounced for the low molecular weight **PVA-L** composite gel compared to **PVA-H**, which may be related to the fact that low molecular weight **PVA-L** is less favorable for recovery to a gel after large deformation.

The falling ball method evaluated the transition temperature from gel to sol during the temperature increase to evaluate the thermal stability of the mixed composite molecular hydrogels. The results (Table 3) showed that the transition temperature of the hydrogels ranged from 40 °C to 60 °C and that they maintained their gel state at about 37 °C, the body temperature of the human body. This suggests that these hydrogels have potential for healthcare applications involving contact with the human body, such as ointment base materials. These results also showed that the transition temperature tended to be lower when the **PVA** had a lower molecular weight. This corresponds to the lower elastic modulus and softer properties of the lower molecular weight **PVA** in the rheometry measurements, which is thought to facilitate the transition due to increased temperature. In addition, for all **PVAs**, the transition temperature tended to decrease when the **PVA** concentration was 2.0 wt.% or higher. This corresponds to the result that, in rheometry measurements, the higher the **PVA** concentration, the more spinnable the gel becomes, and the closer it is to a liquid, the softer and easier it is to flow, which is thought to facilitate the transition when the temperature rises.

**Figure 5:**
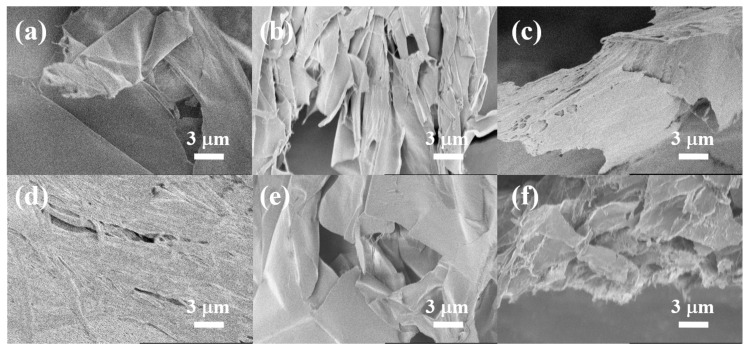
SEM images of the mixed composite and single xerogels and dried sample of **PVA** obtained from freeze-drying of hydrogels and **PVA** aqueous solution: (**a**) xerogel obtained from 1.0 wt.% **NaPPDT** aq.%; (**b**) freeze-dried sample obtained from 8.0 wt.% **PVA-H** aq.; (**c**) xerogel obtained from **NaPPDT**/**PVA-L** 1.0 wt./1.0 wt.%; (**d**) xerogel obtained from **NaPPDT**/**PVA-H** 1.0 wt./1.0 wt.%; (**e**) xerogels obtained from **NaPPDT**/**PVA-L** 1.0 wt./8.0 wt.%; (**f**) xerogels obtained from **NaPPDT**/**PVA-H** 1.0 wt./8.0 wt.%.

Scanning electron microscopy (SEM) observations of the freeze-dried xerogels were used to examine the internal microstructure of the composite gels in the μm to nm range and the surface topography of the constituent elements. From the SEM image in Figure 5, the xerogel of **NaPPDT** alone appeared to be a folded aggregate of several μm wide bands of material that were partially fused together. The dried **PVA** solution also appeared to have overlapping fibrous components of tens of nm width and appeared to be plate-like. The mixed composite xerogels obtained by mixing these materials were found to have finer constituents than the raw material components, particularly the **NaPPDT** xerogels. This suggests that the **NaPPDT** mixed with **PVA** interacted with each other to unwind the aggregates of the **NaPPDT** µm diameter material band into fibrous aggregates of smaller diameters and thicknesses, forming a new fine dense network of which it was a component. This qualitative improvement in the composite gel network due to mixing may be the reason for the improved mechanical properties as measured by dynamic viscoelasticity described above. Although there is a concern that observation of such dried xerogel samples will show artefacts not present in the original gel-like samples due to shape changes during the drying process, it is thought that even if there are changes during the drying process, it is certain that the components have become smaller due to the mixing process as shown in our previous studies [19,20,21].

**Figure 6:**
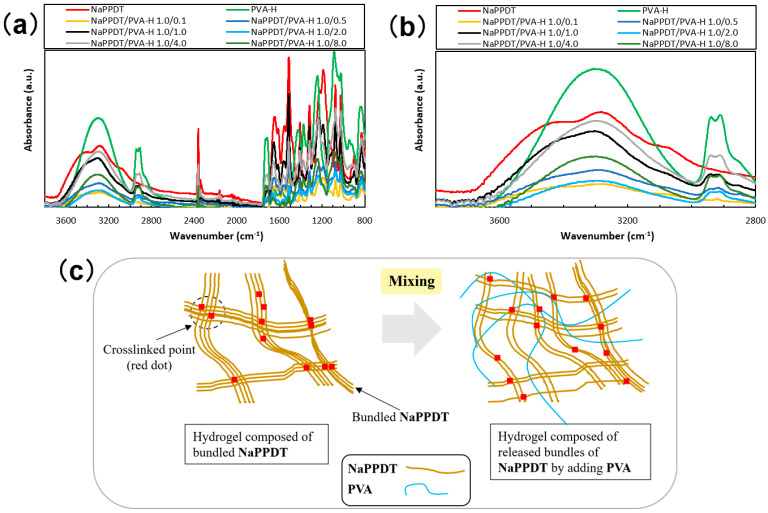
ATR-FTIR spectra of xerogels obtained from **NaPPDT** and composite molecular hydrogels and freeze-dried sample of **PVA-H** (concentration ratios shown as wt.%/wt.%): (**a**) spectra of the entire measurement region; (**b**) spectra of hydroxyl groups and −ONa unit in the stretching vibration region; (**c**) schematic illustrations of a mixed composite hydrogel.

To see the intermolecular interactions between the components in the composite gels, the attenuated total reflectance Fourier transform infrared spectroscopy (attenuated total reflectance (ATR)–FTIR) absorption spectra of the xerogel and the dry samples were evaluated. As depicted in Figure 6a,c, other than the addition of the individual components, no new absorption bands or significant shifts in the absorption bands were observed in the xerogel of the composite gel in the wavenumber region of the stretching vibration of the hydroxyl and sodium sulfonate moieties (3000–2600 cm^−1^). No significant changes were also observed in the absorption region of the amide bonding sites of **NaPPDT**. This is presumably due to the lower concentration of sites contributing to intermolecular interactions involved in mesh formation compared with the concentration of sites involved in absorption bands due to inter- and intramolecular interactions involved for a single component. A schematic illustration of the mesh structure before and after mixing, combining these results with those obtained from rheometry and SEM images, is shown in Figure 6c. As shown in Figure 6c, the mesh of the **NaPPDT** gel was loosened by the addition of the second component, **PVA**, but the mesh was maintained. Further studies on the mesh structure in gels using various composite samples will be necessary in the future.

**Figure 7:**
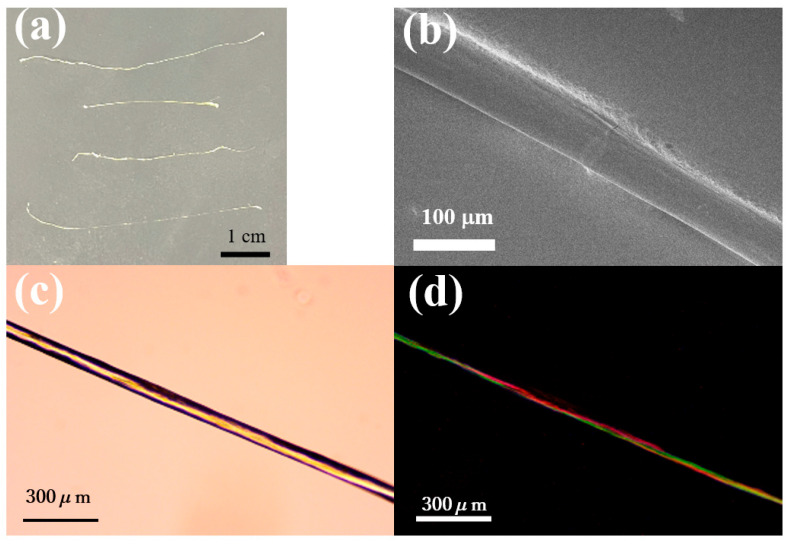
Photograph and images of SEM and polarized light microscope image of the composite gel fiber of **NaPPDT**/**PVA-H** 1.0 wt./8.0 wt.%: (**a**) a photograph of the fiber; (**b**) a SEM image of the fiber; (**c**) a polarized light microscope image of the fiber (without a polarizer); (**d**) a polarized light microscope image of the fiber (under crossed Nicols conditions).

Finally, in order to see the potential application of spinnable hydrogel, an attempt was made to obtain fibrous samples from samples in which spinnable hydrogel was formed by mixing **NaPPDT**/**PVA-L** 1.0 wt.%/8.0 wt.%. For the sample after mixing in the vial, the edges of the gel were pinched with tweezers and pulled to obtain fibrous material. The fibrous material was dried at 60 °C to obtain dried fibers. A photograph, images of SEM, and polarized light microscope of the dried composite gel fibers are shown below. As shown in this Figure 7, straight fibers were obtained in μm order. Polarized light microscopy (under crossed Nicols conditions) showed that the area where the fibers were present appeared bright, indicating that the fibers were oriented or anisotropic. This is thought to be due to the fibers being oriented as a result of drawing to form the fibers and the orientation or anisotropy because **NaPPDT** exhibits lyotropic liquid crystallinity in the mixed concentration range. Thus, it was found that oriented fibers could be obtained from the composite gel. However, as we are still investigating experimental conditions to obtain fiber samples with constant diameter, mechanical property tests including tensile tests of the fibers will be considered in the future.

## 3. Conclusions

A gel-like substance with thixotropic properties was obtained by mixing the polymer hydrogelator **NaPPDT** with the water-soluble polymer **PVA** at low concentrations, where the single component of composites would be a liquid. This is possible with the combination of **NaPPDT** and **PVA** and is a special phenomenon that is not observed with mixtures of aqueous anionic polymer solutions with other sodium sulfonate or phosphonic acid side chains and aqueous **PVA** solutions. In addition, increasing the **PVA** concentration in the composite gel to a high concentration of 2.0 wt.% or more, gel stabilization and the development of spinnable gel properties were observed due to an increase in the gel-sol transition strain, and an increase in the elastic modulus was observed by increasing the molecular weight of **PVA** in the composite gel. These observations can be attributed to the qualitative improvement in the network structure of the gel due to the increased concentration and high molecular weight. Thus, despite the dilution of gelator and **PVA** concentrations when mixing aqueous polymer hydrogelator and **PVA** solutions, composite molecular gel materials with improved mechanical properties could be produced. As this gel material has been shown to be a matrix for functional materials such as **NaPPDT** and **PVA**, respectively, it can be expected to be a potential candidate for a base material for ointments in the healthcare field as a new gel-like matrix.

## 4. Materials and Methods

The polymer hydrogelator **NaPPDT** (M_n_ = 10,000) was prepared according to the previous literature [15] by polycondensation of phenylenediamine and sodium 2-sulfotrerephthalate with LiCl in *N*-methyl-2-pyrrolidone using the phosphorylation method. Pure water was deionized with an Elix UV 3 Milli-Q integral water purification system (Nihon Millipore K.K., Tokyo, Japan). Low molecular weight poly(vinyl alcohol) (average mol wt. 30,000–70,000, 87–90% hydrolyzed), low molecular weight poly(vinyl alcohol) (average M_w_ 146,000–186,000, 87–89% hydrolyzed) poly(sodium 4-styrenesulfonate) (average M_w_ 70,000), and poly(sodium 4-styrenesulfonate) (average M_w_ 1,000,000) were purchased from Sigma-Aldrich Japan (Merck KGaA, Darmstadt, Germany) and used as received. Chondroitin sulfate sodium salt and deoxyribonucleic acid sodium salt were purchased from Tokyo Chemical Industry Co., Ltd. and used as received. All other chemicals were obtained from Wako Pure Chemical Industries, Ltd., Tokyo, Japan, and they were used without purification.

The preparation of **NaPPDT**/**PVA** composite molecular hydrogels was done as follows: at first, 0.5 wt.% and 1.0 wt.% **NaPPDT** aqueous solutions were made by mixing **NaPPDT** solid and pure water and rested for one day at room temperature (the 0.5 wt.% aqueous solution was liquid, and the 1.0 wt.% aqueous solution was gel). Then **PVA** aqueous solutions of various concentrations obtained by dissolving at 90 °C for one day were added to the **NaPPDT** aqueous solutions at 1:1 by weight ratio at room temperature and mixed by use of a vortex genie (Scientific Industries, Inc., Bohemia, New York, NY, USA). Before measurements, the mixed composite hydrogels were rested for 30 min at room temperature.

Gelation and thixotropic properties were determined using the vial inversion method. The vial inversion method judges a mixture as a gel if it does not fall out of the vial containing the mixture when the vial is inverted, and as a sol if it does. The vial inversion method was performed five minutes after the mixture was mixed to detect gelation. The gel was also judged to have returned to gel if the contents did not fall out when the vial was inverted again, after the vial had been left standing following the application of external mechanical force by a vortex mixer to the vial containing the gelled substance, and the gel was judged to have thixotropic properties. The falling-ball method was used to evaluate the change in state from gel to sol with increasing temperature. A 1 mm diameter, 7 mg weight SUS ball was gently placed on the top of the hydrogel in a vial, and the vial was placed on a cool plate CP-085 (Scinics Corporation, Tokyo, Japan) and wrapped with insulation (absorbent cotton), and the temperature of the base plate of the cool plate was raised from 25 °C at 1 °C/min for evaluation. In this study, we employed a Leica ML9300 polarized optical microscope (MEIJI TECHNO CO., LTD., Saitama, Japan) with crossed Nicols to conduct polarized light microscopy observations on composite hydrogels. For SEM image measurements, a JSM-6700FN scanning electron microscope (JEOL Ltd., Tokyo, Japan) operating at 1.0 keV was used. The freeze-dried xerogel samples were carefully positioned on a conductive tape situated on the brass SEM stage. Prior to imaging, a 10 nm thick of Pt was applied to the samples using a sputtering technique to enhance their electrical conductivity. Dynamic rheological measurements for samples were performed using an MCR 302e rheometer (Anton Paar Japan K.K., Tokyo, Japan) with a parallel plate set at a gap of 0.50 mm (8 mm diameter) at 25 °C. Frequency sweeps were conducted with a strain amplitude (γ) of 0.01%, and strain sweeps were carried out at a constant angular frequency of 1 rad s^−1^. For the repeated step-shear measurements, a small strain with an amplitude of 0.01% and a frequency of 1 Hz was applied, followed by a large strain with a shear rate of 3000 s^−1^ for 0.1 s. Furthermore, ATR–FTIR spectra were recorded using a FTIR6600 spectrometer (JASCO Corporation, Tokyo, Japan) in conjunction with a single bounce diamond attenuated total reflectance (ATR) accessory.

## Figures and Tables

**Table 1:** Gelation behavior of mixed solutions of **NaPPDT** aq. and **PVA** aq. (mixing at 1:1 by weight ratio).

	**NaPPDT** 0.1 wt.%	**NaPPDT** 0.5 wt.%	**NaPPDT** 1.0 wt.%
**PVA-L** 0.1 wt.%	L ^1^	L	G ^2^
**PVA-L** 0.5 wt.%	L	L	G
**PVA-L** 1.0 wt.%	L	GD ^3^	G
**PVA-L** 2.0 wt.%	–	–	G
**PVA-L** 4.0 wt.%	–	–	G
**PVA-L** 8.0 wt.%	–	–	G
**PVA-H** 0.1 wt.%	L	L	G
**PVA-H** 0.5 wt.%	L	GD	G
**PVA-H** 1.0 wt.%	L	GD	G
**PVA-H** 2.0 wt.%	–	–	SG ^4^
**PVA-H** 4.0 wt.%	–	–	SG
**PVA-H** 8.0 wt.%	–	–	SG

^1^ L: liquid state. ^2^ G: gel state. ^3^ WG: gel that drips off after 5 min. ^4^ SG: spinnable gel.

**Table 2:** Gelation behavior of aqueous anionic polymer solutions mixed with aqueous **PVA** solutions (mixing at 1:1 by weight ratio).

	PVA-L 1.0 wt.%	PVA-H 1.0 wt.%
**NaPPDT** 1.0 wt.%	G ^1^	G
poly(sodium 4-styrenesulfonate) (M_w_ 70,000) 1.0 wt.%	L ^2^	L
poly(sodium 4-styrenesulfonate) (M_w_ 1,000,000) 1.0 wt.%	L	L
Chondroitin sulfate sodium salt 1.0 wt.%	L	L
Deoxyribonucleic acid sodium salt 1.0 wt.%	L	L

^1^ G: gel state. ^2^ L: liquid state.

**Table 3:** Gel to sol transition temperature of the hydrogels results by falling-ball method.

Mixed Composite Sample	Temperature of Gel to Sol (°C)
**PVA-L** 0.1 wt.%/**NaPPDT** 1.0 wt.%	50 ^1^
**PVA-L** 0.5 wt.% /**NaPPDT** 1.0 wt.%	50
**PVA-L** 1.0 wt.% /**NaPPDT** 1.0 wt.%	50
**PVA-L** 2.0 wt.% /**NaPPDT** 1.0 wt.%	40
**PVA-L** 4.0 wt.% /**NaPPDT** 1.0 wt.%	40
**PVA-L** 8.0 wt.% /**NaPPDT** 1.0 wt.%	40
**PVA-H** 0.1 wt.% /**NaPPDT** 1.0 wt.%	60
**PVA-H** 0.5 wt.% /**NaPPDT** 1.0 wt.%	60
**PVA-H** 1.0 wt.% /**NaPPDT** 1.0 wt.%	61
**PVA-H** 2.0 wt.% /**NaPPDT** 1.0 wt.%	60
**PVA-H** 4.0 wt.% /**NaPPDT** 1.0 wt.%	57
PVA-H 8.0 wt.% /**NaPPDT** 1.0 wt.%	57

^1^ The evaluation was performed by increasing the substrate plate temperature from 25 °C at 1 °C/min.

## Data Availability

Not applicable.
